# Is There Still a Place for Tocilizumab in Coronavirus Disease 2019?

**DOI:** 10.1093/ofid/ofab013

**Published:** 2021-01-12

**Authors:** Timothée Klopfenstein, Thierry Conrozier, N’dri Juliette Kadiane-Oussou, Vincent Gendrin, Souheil Zayet

**Affiliations:** 1 Infectious Disease Department, Nord Franche-Comté Hospital, Trévenans, France; 2 Rheumatology Department, Nord Franche-Comté Hospital, Trévenans, France

**Keywords:** clinical trials, coronavirus, COVID-19, tocilizumab

## Abstract

In this article, we sought to summarize the available evidence of tocilizumab as a treatment for coronavirus disease 2019. Recent tocilizumab randomized trials have not shown clear evidence of efficacy, especially on mortality, in contrast to observational studies. These clinical trials focus on a heterogeneous population of patients (clinical severity and inflammatory stage), and this is possibly one of the reasons that explain heterogeneity of results. However, these same trials have shown some evidence that tocilizumab may reduce intensive care unit admissions and/or mechanical ventilation incidence, which are huge challenges in the severe acute respiratory syndrome coronavirus 2 pandemic. Future clinical trials with primary endpoint built on this assumption are needed (1) to confirm whether tocilizumab reduces mechanical ventilation requirement and (2) to describe the right patient population and optimal timing for tocilizumab administration. Finding the optimal timing for tocilizumab administration and the group of patients who are susceptible to having the greatest benefit are probably the main challenge.

At this time, the second wave of severe acute respiratory syndrome coronavirus 2 (SARS-CoV-2) infection is ongoing in Europe, including France, and we are facing a huge challenge in terms of places in hospitals; intensive care unit (ICU) capacities are especially challenged while facing this outbreak. Until now, corticosteroids have been the only treatment, which have proven to reduce mortality [1]. Indeed, several arguments showed that tocilizumab, “a recombinant humanized anti-interleukin-6 receptor monoclonal antibody”, which is usually used in the treatment of rheumatoid arthritis [2], could be an effective treatment in severe novel coronavirus disease 2019 (COVID-19). By neutralizing a key inflammatory factor in the cytokine release syndrome, this molecule can theoretically block the cytokine storm during the systemic hyperinflammation stage and reduce the severity of the disease [3]. In October 2020, in a cohort of 206 patients with COVID-19, we published a paper showing that tocilizumab can be an effective treatment to reduce mortality and/or invasive mechanical ventilation requirement in COVID-19 [4]. In our study [4], tocilizumab was used in 30 patients in critical condition (oxygen therapy flow at tocilizumab onset was 10.5 L/minute) as a rescue treatment (8 of 30 patients who died were not admitted in ICU in regard to their comorbidities). However, mortality and/or mechanical ventilation requirement were lower in patients with tocilizumab than in 176 patients without tocilizumab (27% vs 52%, *P* = .009). However, since the results of randomized prospective clinical trials [5–7], tocilizumab has disappointed medical community’s hope on this drug due to the lack of impact on mortality (during the first month after administration), in contrast to observational studies. For example, in a randomized clinical trial, Stone et al [5] showed no benefit of tocilizumab versus placebo on mechanical ventilation or death on day 28 in a population at inflammatory stage (median C-reactive protein level at 110.0 mg/L) of moderate to severe pneumonia (>95% of patients had a level of O_2_ <6l/minute delivered by nasal cannula or no O_2_ administration). Likewise, Salvarani et al [6] showed no benefit of tocilizumab early administration in a selected population of severe (but not critical) pneumonia (median PaO_2_/FiO_2_ at 264.5 mmHq), which had a very low level of systemic inflammation (median C-reactive protein at 8.2 mg/L). Recent tocilizumab randomized trials have not shown clear evidence of efficacy, especially on mortality [5–9]. However, these same trials have shown some evidence that tocilizumab may reduce ICU admissions and/or mechanical ventilation incidence. In this way, EMPACTA [8] met its primary endpoint: first results (Carlos Salama, 2020, unpublished observations) have shown that the cumulative proportion (95% confidence interval [CI]) of patients requiring mechanical ventilation or who had died by day 28 was 12.0% (95% CI, 8.52% to 16.86%) and 19.3% (95% CI, 13.34% to 27.36%) for the tocilizumab and placebo arms, respectively (log-rank *P* = .0360; hazard ratio [HR], 0.56; 95% CI, 0.33 to 0.97). The significance of this result is due to a clear decrease in mechanical ventilation incidence in the tocilizumab arm because there were no impact on mortality by day 28: 10.4% (95% CI, 7.2% to 14.9%) in the tocilizumab arm and 8.6% (95% CI, 4.9% to 14.7%) in the placebo arm (weighted difference, 2%; 95% CI, –5.2% to 7.8%). EMPACTA included hospitalized COVID-19 pneumonia patients with SpO_2_ <94% while on ambient air before critical stage (before stage of continuous positive airway pressure or bilevel positive airway pressure or invasive mechanical ventilation). We do not have more detail about patient population. Hermine et al [9] (CORIMUNO-TOCI-1) showed that on day 14, 12% (95% CI, −28% to 4%) fewer patients needed noninvasive ventilation or invasive mechanical ventilation or died in the tocilizumab group compared with the “standard of care” group (24% vs 36%; median posterior HR, 0.58; 90% credible interval, 0.33–1.00), with a posterior probability of HR less than 1 of 95.0%. At baseline, all patients had severe pneumonia with a level ≥3 liters of O_2_ (but no patients on mechanical ventilation). COVACTA’s clinical trial [7], which mainly included severe and critical pneumonia, did not reach the primary endpoint, which was the clinical status on day 28 based on 7-category ordinal scale (1, discharged or ready for discharge; up to 7, death). Statistically, there was no significant improvement for tocilizumab versus placebo (odds ratio 1.0 [95% CI, 1.0 to 1.0] vs 2.0 [95% CI, 1.0 to 4.0], respectively, odds ratio 1.19 [95% CI, 0.81 to 1.76]; *P* = .36). However, clinical status on day 14 improved for tocilizumab versus placebo (odds ratio 3.0 [95% CI, 2.0 to 4.0] vs 4.0 [95% CI, 3.0 to 5.0], respectively, odds ratio 1.42 [95% CI, 0.99 to 2.05]; *P* = .05). This is explained by a lower incidence of ICU stays among patients not in ICU at baseline in the tocilizumab arm compared with the placebo arm (23.6%, n = 30 of 127 vs 40.6%, n = 26 of 64; *P* = .01). These clinical trials (5–9) focus on a heterogeneous population of patients (clinical severity and inflammatory stage); this is possibly one of the reasons that explain heterogeneity of results. However, most of these trials (except Stone et al [5]) have shown some encouragement about secondary endpoints, especially ICU care characteristics. No safety signal for tocilizumab emerged from the 5 clinical trials. All endpoints of the 5 randomized clinical trials are summarized in the Table 1.

**Table 1.** Primary and Other Endpoints on Tocilizumab Randomized Clinical Trials in COVID-19 Patients

			Another Endpoint^b^				
Studies	Study Characteristic^a^	Primary Endpoint	Death	ICU Admission and ICU Characteristics	Clinical Evolution	Duration of Hospitalization	Safety
Stone et al [5] (BACC Bay)	243 patients (161 in TCZ arm)	Mechanical ventilation or death: at day 28, 17 patients (10.6%) in the tocilizumab group and 10 patients (12.5%) in the placebo group had been intubated or had died. HR was 0.83 (95% CI, 0.38–1.81; *P* = .64 by log-rank test). The adjusted HR was 0.66 (95% CI, 0.28–1.52).	Among the 233 patients who were not in the ICU at enrollment, 25 patients (15.9%) in the tocilizumab group and 12 patients (15.8%) in the placebo group were either admitted to the ICU or died before ICU admission.	Duration of mechanical ventilation (days, IQR) was shorter in TCZ group (15–12.6) than placebo group (27.9–16.3) without significant statistical difference	Clinical worsening on ordinal scale	The median time to discharge was 6.0 days in both groups.	Neutropenia developed in 22 patients in the tocilizumab group, compared with only 1 patient in the placebo group (*P* = .002)
	United States				The HR for worsening in the tocilizumab group compared with the placebo group was 1.11 (95% CI, 0.59–2.10; *P* = .73 by log-rank test). The adjusted HR was 0.88 (95% CI, 0.45–1.72).		**Serious infections occurred in fewer patients in the tocilizumab group (13 [8.1%] vs 14 [17.3%]; *P* = .03) **
	Moderate and severe pneumonia						
Salvarani et al [6] (RCT-TCZ-COVID-19)	126 patients (60 in TCZ arm)	Seventeen patients of 60 (28.3%) in the tocilizumab arm and 17 of 63 (27.0%) in the standard care group showed clinical worsening within 14 days since randomization (rate ratio, 1.05; 95% CI, 0.59–1.86; *P* = .87)	Mortality at 14 days (1.7% vs 1.6%; rate ratio, 1.05; 95% CI, 0.07–16.4) and at 30 days (3.3% vs 1.6%; rate ratio, 2.10; 95% CI, 0.20–22.6) was comparable in the 2 groups.	Eleven patients were admitted to ICU, all within 14 days since randomization, with no major differences between the 2 arms (10.0% vs 7.9%, respectively). The rate ratio was 1.26 (95% CI, 0.41–3.91)		The proportion of patients discharged within 14 and 30 days was the same in the 2 groups (rate ratio, 0.99; 95% CI, 0.73–1.35; and 0.98; 95% CI, 0.87–1.09; respectively)	Serious adverse events occurred in 3 patients: 2 (standard care) and 1 (experimental)
	Italy, 24 sites severe pneumonia						
EMPACTA [8]	389 patients (249 in TCZ arm)	**The cumulative proportion (95% CI) of patients requiring mechanical ventilation or who had died by Day 28 was 12.0% (8.52%–16.86%) and 19.3% (13.34%–27.36%) for the tocilizumab and placebo arms, respectively (log-rank *P* = .0360; HR, 0.56 [95% CI, 0.33–0.97]). **	Mortality (95% CI) by day 28 was 10.4% (7.2%–14.9%) in the tocilizumab arm and 8.6% (4.9%–14.7%) in the placebo arm (weighted difference, 2% [95% CI, –5.2% to 7.8%])		Median (95% CI) time to improvement in ordinal clinical status up to day 28 was 6.0 days (6.0–7.0) with tocilizumab and 7.0 days (6.0–9.0) with placebo (HR, 1.15 [95% CI, 0.90–1.48])	Median (95% CI) time to hospital discharge/ready for discharge up to day 28 was 6.0 days (6.0–7.0) with tocilizumab and 7.5 days (7.0–9.0) with placebo (HR, 1.16 [95% CI, 0.91–1.48])	Overall, adverse events were reported in 50.8% of 250 patients and 52.8% of 127 patients in the tocilizumab and placebo arms, respectively, through day 60 and serious adverse events in 15.2% and 19.7%
	United States, South Africa, Kenya, Brazil, Mexico, and Peru.				**Time to clinical failure to day 28 was longer in the tocilizumab arm compared with the placebo arm (median [days]: tocilizumab = NE; PBO = NE; log-rank *P* = .0217; HR [95% CI] = 0.55 [0.33–0.92])**		Through day 60, 5.2% had 16 serious infections (TCZ) and 7.1% had 11 (placebo)
	Severe pneumonia						
Hermine et al [9] (CORIMUNO-TOCI-1)	131 patients (63 in TCZ arm)	On day 4, 12 of 63 (19%) patients randomized to receive TCZ had a WHO-CPS score higher than 5 vs 19 of 67 (28%) in the UC group (median posterior ARD, −9%; 90% CrI, –21 to 3) On day 14, at least 1 event (noninvasive ventilation or mechanical ventilation or died) had occurred in 15 patients in the TCZ group (24%) (cumulative incidence of event 24%; 95% CI, 13%–35%) and 24 patients in the UC group (cumulative incidence 36%; 95% CI, 33%–58%) (posterior median HR, 0.58; 90% CrI, 0.33–1.00)	At day 28, 7 patients had died in the TCZ group and 8 in the UC group (adjusted HR, 0.92; 95% CI, 0.33–2.53).	**Among patients who were not in ICU at randomization, 11 of 60 (18%) in the TCZ group and 22 of 64 (36%) in the UC group were subsequently admitted to the ICU (risk difference, 18%; 95% CI, 0.4%–31%)**	Cumulative incidence of patients who have been weaned from oxygen at day 28 was 89% (95% CI, 78%–95%) and 75% (95% CI, 62%–83%) in the TCZ and UC group, respectively (HR, 1.41; 95% CI, 0.98–2.01)	**The cumulative incidence of discharge by day 28 was 83% (95% CI, 70%–90%) and 73% (95% CI, 61%–82%), respectively (HR, 1.52; 95% CI, 1.02–2.27)**	28 (44%) and 36 (54%) patients in the TCZ and UC groups reported adverse events between randomization and day 28
	France, 9 sites severe pneumonia		The number of patients with mechanical ventilation or death at day 14 was 11 (17%) and 18 (27%) in the TCZ and UC groups. The posterior probability of HR less than 1 and HR less than 0.85 was 92.5% and 84.4%, respectively (posterior median HR, 0.58; 90% CrI, 0.30–1.09)	**Status at day 14**			Serious adverse events occurred in 20 (32%) in the TCZ group and 29 (43%) in the UC group (*P *= .21)
							**The number of serious adverse events was lower in the TCZ than UC group (27 vs 57) with a decreased incidence of serious bacterial infections (2 vs 11)**
COVACTA [7]	444 patients (294 in TCZ arm)	Clinical status at day 28 was not statistically significantly improved for tocilizumab vs placebo (*P* = .36). Median (95% CI) ordinal scale values at day 28: 1.0 (1.0–1.0) for tocilizumab and 2.0 (1.0–4.0) for placebo (odds ratio, 1.19 [0.81–1.76]).	There was no difference in mortality at day 28 between tocilizumab (19.7%) and placebo (19.4%) (difference, 0.3% [95% CI, –7.6 to 8.2]; nominal *P* = .94).	The incidence of mechanical ventilation among patients not mechanically ventilated at randomization was 27.9% (51/183) in the tocilizumab arm and 36.7% (33/90) in the placebo arm (weighted difference, –8.9% [95% CI, –20.7% to 3.0%]; Cochran-Mantel-Haenszel nominal *P* = .14).	**Clinical was improved for tocilizumab versus placebo (*P* = .05). Median (95% CI) ordinal scale values at day 28: 3.0 (2.0–4.0) for tocilizumab and 4.0 (3.0–5.0) for placebo (odds ratio, 1.42 [95% CI, 0.99–2.05]).**	Median time to hospital discharge was 8 days shorter with tocilizumab than placebo (20.0 and 28.0, respectively; nominal *P* = .037; HR 1.35 [95% CI, 1.02–1.79]).	Serious adverse events occurred in 34.9% of 295 patients in the tocilizumab arm and 38.5% of 143 in the placebo arm
	International: 9 countries in Europe and North America			**Median duration of ICU stay was 5.8 days shorter with tocilizumab than placebo (9.8 and 15.5, respectively; nominal *P* = .045).**	Median (95% CI) time to improvement from baseline in ≥2 categories on the 7-category ordinal scale was 14 days (12–17) in the tocilizumab arm and 18 days (15–28) in the placebo arm (log-rank *P* = .08; Cox proportional hazards ratio 1.26 [95% CI, 0.97–1.64])		No tocilizumab-treated patients experienced anaphylaxis
	Severe and critical pneumonia			The median (95% CI) number of ventilator-free days was 22.0 (18.0–28.0) with tocilizumab and 16.5 (11.0–26.0) with placebo (difference, 5.5 [–2.8 to 13.0]; van Elteren *P* = .32)	**Clinical failure (defined by death, withdrawal during hospitalization, transfer to ICU, or requirement for invasive mechanical ventilation within 28 days of baseline) among patients not on mechanical ventilation was lower in TCZ group than placebo group (29% vs 42%, *P* = .03)**		Seventy-six serious infections were reported in 62 patients (21.0%) in the tocilizumab arm and 49 in 37 patients (25.9%) in the placebo arm through day 28
				The incidence of ICU stay among patients not in ICU at baseline was 23.6% (30/127) in the tocilizumab arm and 40.6% (26/64) in the placebo arm (weighted difference, –17.2% [95% CI –31.3% to –3.0%]; *P* = .01).			

Abbreviations: ARD, absolute risk difference; CI, confidence interval; COVID-19, coronavirus disease 2019; CrI, credible interval; HR, hazard ratio; ICU, intensive care unit; IQR, interquartile range; NE, not estimable; PBO, placebo; TCZ, tocilizumab; UC, usual care; WHO-CPS, World Health Organization-Combined Positive Score.

NOTE: Bold represents results with significant differences.

^a^Stone et al [5] , EMPACTA, and COVACTA trials were blinded with placebo controlled (but not Salvarani et al [6] and Hermine et al [9] trials). Clinical severity definition changed by study, so we used the classification of the National Institutes of Health COVID-19 management categories: moderate (SpO_2_ ≥94% on room air) or severe (SpO_2_ <94% on room air) or critical (respiratory failure, septic shock, and/or multiple organ dysfunction).

^b^Stone et al [5], Salvarani et al [6], and COVACTA trials did not reach the primary endpoint. The results of secondary endpoints need to be taken carefully in these studies, *stricto sensu* the difference cannot be considered statistically significant because the primary endpoint was not met.

Finding the optimal, timing of tocilizumab administration and the group of patients who are susceptible to have the greatest benefit are probably the main challenge. For example, in our retrospective cohort [4], patients had a high level of oxygen therapy (mean oxygen therapy flow at tocilizumab onset was 10.5 liters/minute) at a high inflammatory stage (mean serum levels of C-reactive protein and ferritin was 142 mg/L and 1496 ng/mL, respectively, at tocilizumab onset). Our patients were more severe than patients described by Stone et al [5] and Salvarani et al [5] in their cohorts. For example, in Stone et al trial [5], patients were excluded if they were receiving supplemental oxygen ≥10 liters/minute. Our patients were more like CORIMUNO-TOCI-1 patients (oxygen therapy flow ≥3 liter/minute but no patients on invasive mechanical ventilation) [4, 9]. In COVACTA [7], the population was heterogeneous; however, if, at baseline, we selected the patients who were similar to our patients in term of severity (category 4 of the 7-category ordinal scale: ICU or non-ICU hospital ward, requiring high-flow oxygen or noninvasive ventilation), then this category is the only category (among the 7 categories) that has improved their clinical status on day 28 compared with placebo: 1.0 (1.0 to 2.0) for tocilizumab and 2.0 (1.0 to 6.0) for placebo (odds ratio, 1.59 [95% CI, 0.78 to 3.24]). The lack of statistical significance is probably due to the lack of evaluated impact on ICU admissions/characteristic on day 28 by the 7-category ordinal scale (most of the survival patients would be out of ICU) as we discussed above. We do not have the detailed 7-category ordinal scale on day 14 (according to baseline ordinal scale category), which would be interesting. 

## PATIENT CONSENT STATEMENT

Due to the retrospective nature of the study, the Ethics & Scientific Committee of Nord Franche Comté Hospital determined that patients consent was required only for the off-label use Tocilizumab. We made sure to keep patient data confidential and in compliance with the Declaration of Helsinki.

## CONCLUSIONS

To conclude, randomized trials with tocilizumab in COVID-19 pneumonia have not shown an impact on mortality. However, tocilizumab might have some benefit in patients with severe COVID-19 in reducing mechanical ventilation incidence or ICU admissions, which are huge challenges in the SARS-CoV-2 pandemic. A recent meta-analysis about these randomized trials confirmed our conclusion [10]. Furthermore, although tocilizumab had no impact on mortality during the first month (after administration), it may decrease the risk of long-term complications and possibly death by reducing mechanical ventilation incidence or ICU admissions. Future clinical trials with primary endpoint built on this assumption are needed to confirm whether tocilizumab reduces mechanical ventilation requirement and to describe the right population and optimal timing administration with expected benefits of tocilizumab use. If tocilizumab is effective, it is probably on severe/critical COVID-19 pneumonia patients with a high level of oxygen therapy or noninvasive ventilation (before intubation stage).

## References

[1] Horby P, Lim WS, Emberson JR, et al.; RECOVERY Collaborative Group. Dexamethasone in hospitalized patients with Covid-19–preliminary report. N Engl J Med 2020; doi:10.1056/NEJMoa2021436 PMC7383595 32678530

[2] Smolen JS, Beaulieu A, Rubbert-Roth A, et al.; OPTION Investigators Effect of interleukin-6 receptor inhibition with tocilizumab in patients with rheumatoid arthritis (OPTION study): a double-blind, placebo-controlled, randomised trial. Lancet 2008; 371:987–97. 18358926 10.1016/S0140-6736(08)60453-5

[3] Zhang C, Wu Z, Li JW, et al. Cytokine release syndrome in severe COVID-19: interleukin-6 receptor antagonist tocilizumab may be the key to reduce mortality. Int J Antimicrob Agents 2020; 55:105954. 32234467 10.1016/j.ijantimicag.2020.105954 PMC7118634

[4] Klopfenstein T, Zayet S, Lohse A, et al.; HNF Hospital Tocilizumab multidisciplinary team Impact of tocilizumab on mortality and/or invasive mechanical ventilation requirement in a cohort of 206 COVID-19 patients. Int J Infect Dis 2020; 99:491–5. 32798660 10.1016/j.ijid.2020.08.024 PMC7423574

[5] Stone JH, Frigault MJ, Serling-Boyd NJ, et al. Efficacy of tocilizumab in patients hospitalized with Covid-19. N Engl J Med 2020; doi:10.1056/NEJMoa2028836 PMC7646626 33085857

[6] Salvarani C, Dolci G, Massari M, et al. Effect of tocilizumab vs standard care on clinical worsening in patients hospitalized with COVID-19 pneumonia: a randomized clinical trial. JAMA Intern Med 2020; doi:10.1001/jamainternmed.2020.6615 PMC7577199 33080005

[7] Furlow B COVACTA trial raises questions about tocilizumab’s benefit in COVID-19. Lancet Rheumatol 2020; 2:e592. 10.1016/S2665-9913(20)30313-1 PMC7480990 32929415

[8] Salama C, Han J, Yau L, et al. Tocilizumab in Patients Hospitalized with Covid-19 Pneumonia. N Engl J Med 2021; 384:20–30. 33332779 10.1056/NEJMoa2030340 PMC7781101

[9] Hermine O, Mariette X, Tharaux P-L, et al. Effect of tocilizumab vs usual care in adults hospitalized with COVID-19 and moderate or severe pneumonia: a randomized clinical trial. JAMA Intern Med 2020; doi:10.1001/jamainternmed.2020.6820 PMC7577198 33080017

[10] Tleyjeh IM, Kashour Z, Damlaj M, et al. Efficacy and safety of tocilizumab in COVID-19 patients: a living systematic review and meta-analysis. Clin Microbiol Infect 2020; doi:10.1016/j.cmi.2020.10.036 PMC7644182 33161150

